# SparkGA2: Production-quality memory-efficient Apache Spark based genome analysis framework

**DOI:** 10.1371/journal.pone.0224784

**Published:** 2019-12-05

**Authors:** Hamid Mushtaq, Nauman Ahmed, Zaid Al-Ars

**Affiliations:** Quantum and Computer Engineering, Delft University of Technology, Delft, The Netherlands; Oklahoma State University, UNITED STATES

## Abstract

Due to the rapid decrease in the cost of NGS (Next Generation Sequencing), interest has increased in using data generated from NGS to diagnose genetic diseases. However, the data generated by NGS technology is usually in the order of hundreds of gigabytes per experiment, thus requiring efficient and scalable programs to perform data analysis quickly. This paper presents SparkGA2, a memory efficient, production quality framework for high performance DNA analysis in the cloud, which can scale according to the available computational resources by increasing the number of nodes. Our framework uses Apache Spark’s ability to cache data in the memory to speed up processing, while also allowing the user to run the framework on systems with lower amounts of memory at the cost of slightly less performance. To manage the memory footprint, we implement an on-the-fly compression method of intermediate data and reduce memory requirements by up to 3x. Our framework also uses a streaming approach to gradually stream input data as processing is taking place. This makes our framework faster than other state of the art approaches while at the same time allowing users to adapt it to run on clusters with lower memory. As compared to the state of the art, SparkGA2 is up to 22% faster on a large big data cluster of 67 nodes and up to 9% faster on a smaller cluster of 6 nodes. Including the streaming solution, where data pre-processing is considered, SparkGA2 is 51% faster on a 6 node cluster. The source code of SparkGA2 is publicly available at https://github.com/HamidMushtaq/SparkGA2.

## Introduction

DNA sequence analysis has become an important tool used in many kinds of applications from forensics to medicine. The data is usually sequenced using next generation sequencing (NGS) machines, which produce oversampled data, resulting in a large amount of data to process by sequence analysis programs. The size of the whole human genome data produced by these machines is in the range of hundreds of GBs. In order to process this data fast, we need large amounts of computational resources.

In this paper, we propose a new Apache Spark [1] based framework called SparkGA2 that allows the GATK best-practices pipeline [2] to run efficiently and cost-effectively on a scalable computational cluster. SparkGA2 uses Spark’s in-memory computation capabilities to improve the performance of the framework. We also use compression techniques to lower the memory footprint. In addition, we can configure the framework to require less system memory at the cost of increased runtime. Due to this feature, it can even be run on clusters with limited available RAM. Moreover, it uses the pipeline tools unmodified, meaning that one tool can easily be replaced with another one.

In the following, we list the contributions of this paper.

- We implemented an on-the-fly compression method of intermediate data that allows us to decrease the memory footprint by up to 3x.
- SparkGA2 is able to trade off performance at the expense of system resource utilization, allowing higher speed with more memory, while still allowing proper functionality with lower amounts of memory.
- By improving the efficiency of data access, we reduce reliance on the Hadoop file system and improve performance as compared to state-of-the-art tools, such as SparkGA [3].
- We ensured a modular implementation of the framework as three distinct steps that allow us to optimize the Spark runtime parameters for each step.

This paper is organized as follows. In Section **Background**, we discuss the different stages of the GATK best-practices pipelines and related work. Section **Methods** presents the Apache Spark framework we implemented to enable pipeline scalability. This is followed by Section **Results and discussion**, which discusses the performance and accuracy evaluations. We finally conclude the paper with Section **Conclusions**.

## Background

In this section, first we discuss the GATK best-practices pipeline and afterwards discuss related work.

### GATK best-practices pipeline

DNA analysis is done with raw over-sampled sequencing reads obtained from a DNA sequencing machine. Due to oversampling, the sequenced data is quite large, usually in the range of 100s of GB, for the whole genome of a human. A standard way of storing such raw sequenced reads is the FASTQ file format [4].

GATK best-practices pipeline [2], is an example of a DNA analysis pipeline, where using tools like Bowtie2 [5] or Burrows-Wheeler Aligner (BWA mem) [6], reads are mapped to the corresponding positions of the human genome using a reference genome. Afterwards, multiple copies of the same raw reads are marked as duplicates, by using Picard tools for example. Afterwards, several more steps are performed, such as performing local realignment of reads around indels (Indel realignment), adjusting quality scores of mapped reads (Base recalibration) and discovering all the variants (Haplotype caller), before we get the final output in a VCF file.

In the pipeline, some of the tools scale very well, such as the mapping tools, while the other tools do not scale as well. Besides that, even the mapping tool cannot run in a distributed fashion on a cluster. Therefore, we have developed a generic framework, which can distribute such computation on a cluster. We achieve that with the help of Apache Spark.

### Related work

One of the earliest big data based DNA analysis frameworks is Churchill [7], which utilizes the available computational resources using a number of proprietary tools in a tightly-integrated computational pipeline. However, the proprietary closed-source nature of the pipeline makes it inaccessible in practice.

Another example is Halvade [8], which uses the Hadoop MapReduce framework and allows the original unmodified GATK pipeline tools to be used. The pipeline execution is divided into three main parts. First, parallel map tasks are used to perform DNA mapping, the output of which is collected as <chromosomal region, mapped read> key-value pairs. Then a sorting step is performed to reorder the mapped reads according to their location in the genome. The rest of the pipeline is then executed by a set of reduce tasks performing mark duplicates, base recalibration and haplotype calling, and resulting in an output VCF file.

Since Halvade uses the MapReduce framework, it performs the various steps in the pipeline primarily in disk rather than in memory. This causes an unnecessary disk access penalty as a result of the large genomics data sets used in practice. Secondly, it creates chromosomal regions based on the length of the chromosomes, while not checking the actual number of reads in each region. This causes some regions to become disproportionately larger than others resulting in high bottlenecks in the execution of all regions, as the framework waits for the largest region to finish.

A couple of efforts [9, 10] address the problems with MapReduce based solutions by using a Spark based implementation of the GATK best-practices pipeline. [10] processes the DNA pipeline fully in memory, and thus is able to create chromosomal regions based on the number of reads. However, since its load balancing step is done fully in memory, this results in out of memory errors for large input files. In addition, it does not differentiate between the requirements of the various stages of the GATK pipeline, and runs the whole pipeline as a single run, thereby not fully optimizing system utilization for each stage. [9] on the other hand does not have its source code publicly available.

As a followup to [10], SparkGA [3] implements a load balancing step that is more memory efficient. In addition, it runs the program in 3 different steps: DNA mapping & static load balancing; sorting & dynamic load balancing; and marking of duplicates and variant discovery. This allows SparkGA to configure each step separately to optimize resource utilization and overall performance. This solution reduces the memory requirements of the load balancing step by dividing the genome into regions and only loading each region into memory one at a time. One drawback of this approach is that it creates too many files after the mapping step, all of which need to be uploaded to HDFS. This significantly impacts the performance on commercial cloud services where access to HDFS can be slow.

SparkGA2, on the other hand, can adapt the number of files created depending on the available memory in the cluster, which allows for trading off performance for memory requirements. In addition, we reduce the memory footprint by compressing the data before copying it into the memory.

## Methods

We created SparkGA2 based on the Apache Spark framework such that it can parallelize the execution of the various stages of the GATK pipeline. Most of the tools used in GATK are executed in SparkGA2 unchanged with the exception of “sorting” and “SAM to BAM conversion”. These tools are replaced with native Spark implementations within our framework. A summary of the used tools is listed in Table 1.

**Table 1:** Comparison of tools used in GATK best-practices pipeline and SparkGA2.

Step	GATK	SparkGA2
**Align reads**	BWA mem	BWA mem
**SAM to BAM**	Picard	Picard’s Java library
**Sort reads**	Picard	Sorting in Scala
**Mark duplicates**	Picard	Picard
**Indel realignment**	GATK	GATK
**Base recalibration**	GATK	GATK
**Haplotype caller**	GATK	GATK

In the following, we first discuss the overview of our optimized parallel GATK approach, followed by our mapping and compression approach. Next, we discuss our sorting and system resource tradeoff methods, followed by our method to discover the variants.

### Overview

**Algorithm 1** Mapping algorithm

**procedure** DNAMapperRun(fileName)

⊳ **RDD is of type** <**binID**, **content**>

  *mappedInfo* ← *runMapper*(*fileName*)

⊳ **Calculate number of reads and compress each bin**. **RDD is of type** <**binID**, **numReads**, **compressedContent**>

  *compressedBins* ← *compressBins*(*mappedInfo*)

⊳ **Write bins to the HDFS**

  *writeBinsToHDFS*(*compressedBins*, *mapperOutFolderPath*)

⊳ **Return information**. **RDD is of type** <**binID**, **numReads**>

  *retArray* ← *compressedBins*.*map*(*x* => (*x*._1, *x*._2))

⊳ **This RDD simply contains the name of the input fastq chunk files**

  *inputData* ← *sc*.*parallelize*(*getFilesList*(*inputFolderPath*))

⊳ **RDD is of type** <**binID**, **numReads**>

  *bwaOut* ← *inputData*.*flatMap*(*fileName* => *DNAMapperRun*(*fileName*))

  *bwaOutStr* ← *bwaOut*.*map*(*x* => *x*._1 + “:” + *x*._2)

  *bwaOutStr*.*saveAsTextFile*(*outputFolderPath* + “*binsInfo*”)

**Fig 1:**
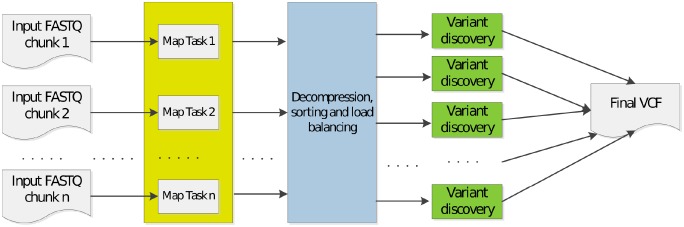
Data flow of SparkGA2.

The dataflow of the execution of SparkGA2 is shown in Fig 1. In a typical DNA sequencing experiment, two FASTQ files are generated, which represent the two ends of a pair of sequences. These two input FASTQ files are divided into interleaved input FASTQ chunks using a chunk segmentation tool, which also uploads these files to HDFS. Each DNA mapping task takes such a FASTQ chunk as an input and aligns its reads using BWA mem, producing a SAM file that contains the mapped reads in the form of SAM records.

The output in our case is a compressed output file containing mapped reads (represented as SAM records) in compressed form, along with some more information. The load balancer would decompress those files and extract the SAM records from them, before putting those SAM records into their corresponding regions. Afterwards, it would sort the SAM records in those regions, before writing them to BAM files, which would then be processed by the last stage of variant discovery.

The last step is done in a single task for each chromosomal region. Each of these tasks produces a VCF file, all of which are then combined at the end to produce the final VCF file. Unlike [3], where a lot of intermediate files are produced between the first and second step, here we only produce as many files as the number of input FASTQ chunks. This greatly reduces access to the HDFS between those two stages.

Moreover, our framework allows input data, which is in gzipped FASTQ format, to be streamed directly to the HDFS for the data nodes to start processing. The details of this streaming mechanism are explained in [11].

We perform the execution in three different steps: 1. mapping; 2. decompression, sorting, and load balancing; and 3. variant discovery. This approach allows us to configure the parameters of each step separately to optimize its performance.

### Step 1: Mapping and resource tradeoffs

**Fig 2:**
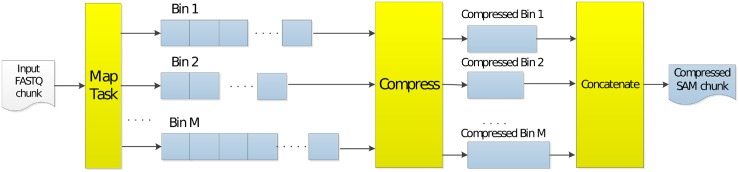
Mapping output in SparkGA2.

The first step of the implementation is the mapping step. Algorithm 1 describes this step, which is illustrated in Fig 2. In this step, for each input FASTQ chunk, a mapping task, such as BWA mem, is executed. The tasks map short reads to a reference and output this information in the form of SAM records, which are then grouped into bins. Bins here represent parts of chromosomes, according to their positions. For example, if we set the bin size to 5000, it would mean that all SAM records in bin 1 of chromosome 1 would have positions from 0 to 4999 of chromosome 1, while bin 2 would have positions from 5000 to 9999. Once BWA is finished, all these bins are then compressed in parallel, and then written to a file. For each bin, the file contains information about the bin’s region and the length of its content, so that the next step can extract them.

Besides these output files, a hash-map is also stored whose keys are binIDs and values are the number of reads in each bin. This information is later used in the load balancing step. Note that the larger the bin size is, the more compact this hash-map becomes. With a bin size of 5000, the size of the hash-map is just a few megabytes.

**Algorithm 2** Load balancing

**procedure** LoadBalancer(binsInfoFolderPath)

⊳ **RDD is of type** <**binID**, **numReads**>

  *binsInfo* ← *getBinsInfo*(*binsInfoFolderPath*)

⊳ **Sum up the number of reads**. **RDD is of type** <**binID**, **numReads**>

  *numReadsPerBin* ← *binsInfo*.*reduceByKey*(_+ _).*sortByKey*().*cache*()

⊳ **Sum up the number of reads**. **RDD is of type** <**binID**, **numReads**>

  *avgReadsPerRegion* ← *numReadsPerBin*.*map*(_._2).*reduce*(_+ _)/*numRegions*

⊳ **Sum up the number of reads**. **RDD is of type** <**binID**, **numReads**>

  *regionsMap* ← *makeRegionsMap*(*numReadsPerBin*, *avgReadsPerBin*)

⊳ **This RDD simply contains the name of the files made by the DNA mapper part**

  *inputData* ← *sc*.*parallelize*(*getInputFilesList*(*mapperOutFolderPath*))

⊳ **This RDD is of type** <**regionID**, **compressedReads**>

  *compressedRegionReads* ← *inputData*.*flatMap*(*x* => *getCompressedReads*(*x*, *regionsMap*)

⊳ **This RDD is of type** <**regionID**, **Array**[**compressedReads**]>

  *compressedRegions* ← *compressedRegionReads*.*mapValues*(*x* => *Array*(*x*)).*reduceByKey*((*a*, *b*) => *a* + +*b*)

⊳ **Finally build the bam and bed files for each region after decompressing the contents**

  *compressedRegions*.*foreach*(*buildBAMandBEDFiles*(_._1, _._2)

**Fig 3:**
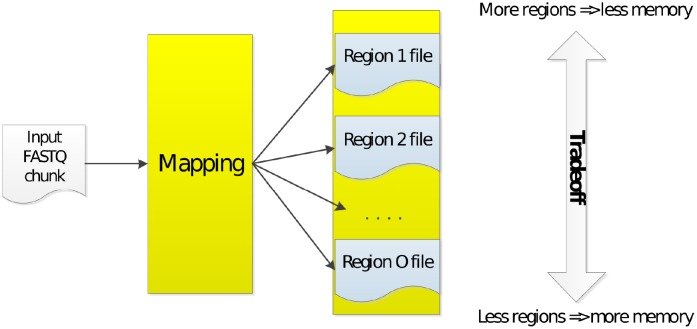
Regions and tradeoff.

Our mapping approach is able to trade off performance at the expense of system resources, allowing higher speed with more memory, while still allowing proper functionality with lower amounts of memory. This approach is illustrated in Fig 3, where each map task is able to generate one or multiple SAM chunks for each input FASTQ chunk. For clusters with low amounts of memory, we can store multiple output files for each map task. This allows processing of smaller files in subsequent pipeline steps such as the load balancing step. For example, we can identify two genome regions by dividing the whole genome into 2 parts, which will reduce the memory requirements of the load balancing step by about half, at the expense of a small extra initialization overhead. The files containing mapped reads that belong to chromosomes from Region 1 would be saved in a specific folder, while the chromosomes from Region 2 in another folder. The load balancing step would then be run twice, each time for a different region. This procedure could improve overall performance on clusters with less memory, because in that way less data is spilled onto the disk.

The input to our SparkGA2 framework is either a single end gzipped FASTQ file or a paired end gzipped FASTQ files. It has to be noted that creating chunks from these gzipped files takes a significant amount of time. Due to this reason, our framework allows data to be streamed directly to HDFS, so that data nodes can start processing data as soon as some data is available. This streaming approach can be done either from URLs directly or gzipped files found on the local file system of the master node. The mechanism of this streaming is explained in detail in [11].

### Step 2: Sorting and load balancing

The process of doing this step is shown in Algorithm 2. Initially, the files which contains the information about number of reads produced for each bin are read. Notice that each mapper task would have created its own such file, so we have to collect this information together by summing the number of reads together for each bin. This is done by using Spark’s *reduceByKey* method. Afterwards, we calculate the average number of reads per region, by dividing the total number of reads in the whole genome by the number of regions that have to be created. Here, the total number of reads is calculated by summing up the reads of all bins. With this information, we can identify the regions in which the different bins are located. We can do this using the *makeRegionsMap* function in Algorithm 2, which takes the number of reads in each bin, and the average number of reads per region. This function would assign the appropriate region to each bin, and return the regions map containing this information.

Once regions map is created, each output file from the mapping step is read in parallel, through the *getCompressedReads* function. This function takes the regions map as input, and assigns a SAM record to the appropriate region. The output is flat mapped, and we get an RDD, whose key is regionID and value is the compressed reads belonging to that region. These compressed reads are then accumulated in arrays, so that we have an RDD, where the key is the region ID and value is an array of compressed SAM records. Finally, this is given to the function *buildBAMandBEDFiles*, which would decompress the SAM records for a region, sort them, and write them onto a BAM file. Besides a BAM file, a BED file is created as well. A BED file here is used to tell the tools in the next step to only work on the region specified in that BED file.

### Step 3: Variant discovery

In this step, the remaining tools in the GATK pipeline are executed using the BAM and BED files generated in Step 2. Each task takes a BAM and BED file as input and performs mark duplicates, base recalibration and haplotype calling to generate a VCF file. The VCF files generated by all the tasks in Step 3 are used to create a combined VCF file. In this step, three tools are used to perform variant discovery.

## Results and discussion

We tested the results on the Microsoft cloud with different number of nodes. Each node contains an 8 core processor with 56 GB of RAM. We used the best practices GATK pipeline from the Broad institute to compute the results. We used BWA version 0.7.10, and GATK version 4.

We also tested our framework on the high performance big data cluster provided by SURFsara (the Dutch HPC infrastructure), with 6, 24, 48 and 67 nodes. Each node on the SURFsara cluster has 56 GB of RAM with 8 cores. On the SURFsara cluster, we used BWA version 0.7.10, and GATK version 3.4 instead of GATK version 4, since the Java run time version found on the data nodes of the SURFsara cluster did not support running GATK version 4.

For both experiments, we tested and compared SparkGA2 with SparkGA, using the publicly available NA12878D dataset (https://allseq.com/knowledge-bank/1000-genome/get-your-1000-genome-test-data-set/), which is 150bp paired-end WGS data having a total size of 272 GB. Moreover, for SURFsara, we used two more benchmarks, the ERR194147 dataset (ftp://ftp.sra.ebi.ac.uk/vol1/fastq/ERR194/ERR194147) which has a total size of 395 GB, and the ERR194160 dataset (ftp://ftp.sra.ebi.ac.uk/vol1/fastq/ERR194/ERR194160) which has a total size of 389 GB, to compare performance of SparkGA2 with SparkGA on 67 nodes.

First we discuss the scalability of our approach followed by a comparison with SparkGA. Lastly, we discuss the memory size vs performance tradeoff capabilities of our framework.

### Scalability

Table 2 lists the runtime of SparkGA2 on the Microsoft cloud with 4, 5 and 6 nodes. The table shows the breakdown of the total runtime for each of the 3 steps of the pipeline in terms of minutes as well as in percentages. The table shows that the performance improves significantly with more number of nodes, which decreases from 531 minutes on 4 nodes down to 344 minutes on 6 nodes. This translates to a reduction of runtime by 54% from 4 to 6 nodes, illustrating the linear scalability of the framework. We note that the mapping step has the highest execution time of up to 56% of the whole pipeline, making it the most appropriate step to optimize performance. It is also interesting to note that each step of the pipeline scales almost equally with the increasing size of the cluster, thereby keeping the same ratios of execution time for each step.

**Table 2:** Runtime of SparkGA2 on Microsoft cloud.

Step	4 nodes	5 nodes	6 nodes
	(mins, %)	(mins, %)	(mins, %)
Step 1	292, **55**%	222, **53%**	192, **56%**
Step 2	79, **15%**	66, **16%**	48, **14%**
Step 3	160, **30%**	129, **31%**	104, **30%**
**Total**	531, **100%**	417, **100%**	344, **100%**

Table 3 shows the runtime of SparkGA2 on the SURFSara cluster with 6, 24, 48 and 67 nodes. Here, we can also see significant improvement in performance as the number of nodes increases, where the total runtime is reduced from 821.5 minutes down to 87 minutes as the cluster scales up from 6 to 67 nodes. This results in a speedup of about 9.4x on the 67 node cluster compared to the 6 node cluster, which is slightly sub-linear compared to the expected 11.2x linear speedup. The relative percentage of the various pipeline steps stays similar as the cluster size increases, indicating little difference in the way the steps scale up. However, there is a slight decrease in the percentage of Step 1 (mapping), indicating better scalability capabilities of BWA. At the same time, we notice the small increase in the percentage of Step 2 (sorting and load balancing), since this step requires heavy network utilization, which becomes a bigger bottleneck as the size of the cluster increases.

**Table 3:** Runtime of SparkGA2 with NA12878 on the SURFSara cluster.

Step	6 nodes	24 nodes	48 nodes	67 nodes
	(mins, %)	(mins, %)	(mins, %)	(mins, %)
Step 1	331.5, **40%**	72.5, **38**%	38.5, **35%**	29, **34%**
Step 2	67.5, **8%**	15.5, **8%**	11, **10%**	10, **11%**
Step 3	422.5, **51%**	101, **53%**	62, **56%**	47, **54%**
**Total**	821.5, **100%**	189, **100%**	111.5, **100%**	87, **100%**

It is interesting to note that the total runtime of the 6-node cluster on SURFSara is 2.4x slower compared to the 6-node Microsoft cloud cluster. This can be attributed to two main reasons: 1. GATK version 4 (on Microsoft cloud) is much faster than the older GATK version 3.4, and 2. the processors used in the SURFSara cluster are slower than their Microsoft cloud counterparts. We also note that Step 3 consumes up to 56% of runtime on SURFSara, again as a result of a slower GATK version 3.4, which makes Step 3 the bottleneck in this case.

### Comparison with SparkGA

**Fig 4:**
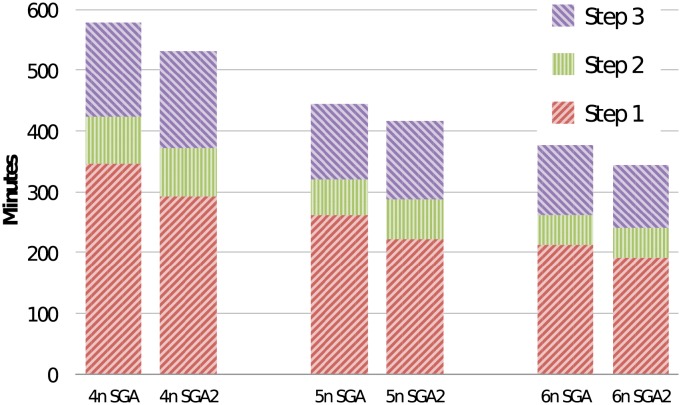
Performance comparison with SparkGA on the Microsoft cluster.

Table 4 shows a comparison of SparkGA2 with SparkGA. The table lists a comparison of runtime for each of the three pipeline steps of the two frameworks for 4, 5 and 6 nodes on the Microsoft cloud, in terms of minutes (m) and relative improvement (imp). The performance comparison is also shown in Fig 4. Each column in the figure is divided into 3 segments representing Step 1, 2 and 3 of the pipeline from bottom to top of each column. The figure shows that the total runtime of both frameworks decreases linearly with an increasing number of nodes in the cluster. However, SparkGA2 is up to 9% faster than SparkGA on this cluster.

**Table 4:** Comparison of SparkGA2 (SGA2) vs SparkGA (SGA) on Microsoft cloud with 4, 5 and 6 nodes.

Step	4 nodes			5 nodes			6 nodes		
	SGA (m, %)	SGA2 (m, %)	imp	SGA (m, %)	SGA2 (m, %)	imp	SGA (m, %)	SGA2 (m, %)	imp
Step 1	347, **60%**	292, **55**%	**18%**	261, **59%**	222, **53%**	**18%**	212, **56%**	192, **56%**	**10%**
Step 2	76, **13%**	79, **15%**	**-4%**	60, **13%**	66, **16%**	**-10%**	51, **14%**	48, **14%**	**6%**
Step 3	155, **27%**	160, **30%**	**-3%**	124, **28%**	129, **31%**	**-4%**	113 **30%**	104, **30%**	**9%**
**Total**	578, **100%**	531, **100%**	**9%**	445, **100%**	417, **100%**	**7%**	376, **100%**	344, **100%**	**9%**

Furthermore, Step 1 of SparkGA2 is up to 18% faster than the corresponding Step 1 in SparkGA. This shows the advantage in performance gained using the two new techniques in Step 1 of SparkGA2: 1. the on-the-fly compression approach which allows for more efficient in-memory data processing, and 2. the reduction in the amount of data uploaded to HDFS during the mapping step.

**Fig 5:**
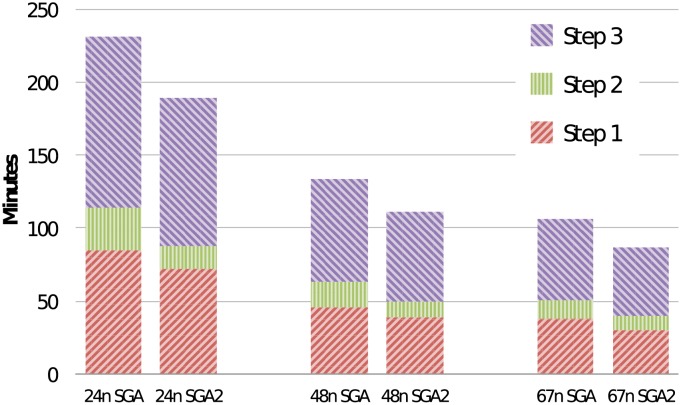
Performance comparison with SparkGA on the SURFsara cluster.

Table 5 shows the results on the SURFsara cluster, where we ran our pipeline with 6, 24, 48 and 67 nodes. The results show that the total runtime of SparkGA2 is 22% faster than SparkGA on a 67 node cluster, with all stages of the pipeline showing an increase in performance. This indicates the impact of the optimizations in SparkGA2 on the total runtime. It is interesting to note that Step 2 shows a particularly high improvement in performance due to the optimized load balancing methods we used. For 24, 48 and 67 nodes, the SparkGA2 load balancer is 87%, 55% and 30% faster than the SparkGA load balancer, respectively. The performance comparison is also shown in Fig 5 for 24, 48 and 67 nodes.

**Table 5:** Comparison of SparkGA2 vs SparkGA on SURFsara cluster with 6, 24, 48 and 67 nodes, for NA12878.

Step	6 nodes			24 nodes			48 nodes			67 nodes		
	SGA (m, %)	SGA2 (m, %)	imp	SGA (m, %)	SGA2 (m, %)	imp	SGA (m, %)	SGA2 (m, %)	imp	SGA (m, %)	SGA2 (m, %)	imp
Step 1	316, **38%**	331.5, **40%**	**-5%**	85, **37%**	72.5, **38%**	**17%**	46, **34%**	38.5, **35%**	**19%**	37.5, **35%**	30, **34%**	**25%**
Step 2	95, **11%**	67.5, **8%**	**41%**	29, **13%**	15.5, **8%**	**87%**	17, **13%**	11, **10%**	**55%**	13, **12%**	10, **11%**	**30%**
Step 3	419, **50%**	422.5, **51%**	**-1%**	117.5, **51%**	101, **53%**	**16%**	70.5, **53%**	62, **56%**	**14%**	55.5, **52%**	47, **54%**	**18%**
**Total**	830, **100%**	821.5, **100%**	**1%**	231.5, **100%**	189, **100%**	**22%**	133.5, **100%**	111.5, **100%**	**20%**	106, **100%**	87, **100%**	**22%**

It is also interesting to note that the total runtime in SparkGA2 shows an increased performance advantage compared to SparkGA as the cluster size increases. This indicates that SparkGA2 has better scalability characteristics, while still being able to run on a small cluster, making it suitable for usage in a practical production environment.

Table 6 shows comparison of SparkGA2’s performance with that of SparkGA for different benchmarks on 67 nodes. We can see that there is clear improvement for all the benchmarks for Step 1 and 2. Step 2 is as fast as 50% for one of the benchmark (ERR194160). Overall, SparkGA2 is up to 22% faster than SparkGA.

**Table 6:** Comparison of SparkGA2 vs SparkGA on SURFSara cluster with 67 nodes, with different benchmarks.

Step	ERR194147			ERR194160			NA12878		
	SGA (m, %)	SGA2 (m, %)	imp	SGA (m, %)	SGA2 (m, %)	imp	SGA (m, %)	SGA2 (m, %)	imp
Step 1	41, **36%**	35, **34**%	**17%**	43.5, **39%**	34, **34%**	**28%**	37.5, **35%**	30, **34%**	**25%**
Step 2	14.5, **13%**	10, **10%**	**45%**	15, **13%**	10, **10%**	**50%**	13, **12%**	10, **11%**	**30%**
Step 3	57.5, **51%**	58.5, **56%**	**-2%**	54, **48%**	56, **56%**	**-4%**	55.5 **52%**	47, **54%**	**18%**
**Total**	113, **100%**	103.5, **100%**	**9%**	112.5, **100%**	100, **100%**	**12.5%**	106, **100%**	87, **100%**	**22%**

**Fig 6:**
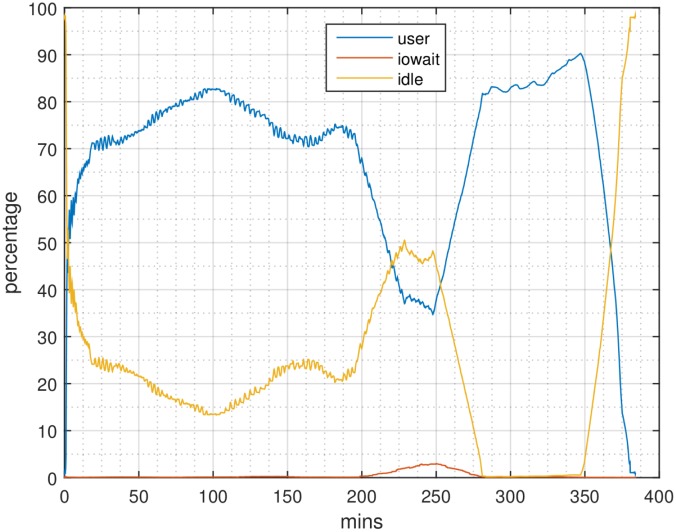
Profile of a node with SparkGA on the 6-node Microsoft cloud cluster.

**Fig 7:**
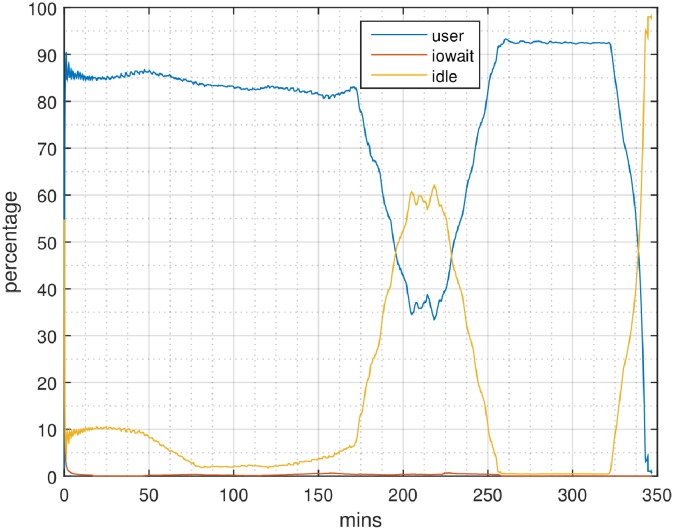
Profile of a node with SparkGA2 on the 6-node Microsoft cloud cluster.

Profiling results for SparkGA and SparkGA2 on the 6-node Microsoft cloud cluster are shown in Figs 6 and 7, respectively. In those figures, the x-axis represents time and the y-axis represents the percentage of resource utilization. CPU usage is expressed in terms of user time and idle time, while I/O usage in terms of io wait time. From those figures we can see that for Step 1, the idle time in the case of SparkGA is much higher than in the case of SparkGA2. The reason is that SparkGA has to upload a lot more files to HDFS as compared to SparkGA2. For Step 2, the profile of SparkGA shows an increase in disk access, causing io wait time as compared to a negligible io wait time for SparkGA2. This can be explained by the large number of files that need to be created and accessed in SparkGA for each of the genome regions created in Step 1, which creates I/O access conflicts.

Table 7 compares the runtime of SparkGA and SparkGA2 on the 6-node Microsoft cloud using the streaming solution for input data. Streaming of input data eliminates the need to spend execution time separately on uncompressing the input FASTQ files, making smaller input chunks for mapping, and uploading them to the HDFS. This is done by a chunking utility, which performs chunking in parallel to the mapping. The chunking utility here is run on the master node. When streaming of input data is taken into account, SparkGA2 is 51% faster than SparkGA, due to SparkGA2’s ability to have input data streamed directly to the HDFS, while data nodes are processing that data. In this way, it can completely overlap such chunking with mapping.

**Table 7:** Runtime in minutes using the streaming approach on Microsoft cloud with 6 nodes.

Step	SparkGA	SparkGA2
	mins	mins
Chunking	143.5	-
Step 1	212	192
Step 2	51	48
Step 3	113	104
**Total**	**520**	**344**

### Efficient memory utilization

We also checked the impact of our approach to trade off performance with system resource utilization in the mapping step, specifically the amount of memory utilization as a result of increasing the number of genome regions. The maximum memory needed to store the regions in-memory is dependent on the number of genome regions in the pipeline. Table 8 lists the amount of memory consumed for 1 to 10 genome regions. The table shows that memory requirements decrease from 122 GB with 1 region down to 20 GB with 10 regions. This indicates that by increasing the number of regions, we can significantly reduce the memory requirements of the cluster, making our framework executable even for a smaller cluster (albeit with an increased runtime).

**Table 8:** Maximum memory consumed by number of regions.

**No**. **of regions**	**1**	**2**	**4**	**6**	**8**	**10**
**Memory**	122 GB	69 GB	37 GB	29 GB	20 GB	20 GB

The impact on runtime is shown in Table 9, which lists the runtime results of Step 2 (sorting and load balancing) for the 6-node Microsoft cluster. The table shows that the total time for Step 2 increases with increasing number of regions, from 48 minutes (6 regions) up to 56 minutes (10 regions), due to the startup overhead for Spark for each of the regions. For lower than 6 regions, the cluster is not able to run the pipeline, due to the increased memory consumed by Spark for storing region data, causing the cluster to go out of memory.

**Table 9:** Runtime in minutes for Step 2 on Microsoft cloud with 6 nodes, with different number of regions.

**No**. **of regions**	**6**	**8**	**10**
**Time**	48 min	51 min	56 min

## Conclusions

This paper proposed SparkGA2, a production quality, general purpose Spark framework which can run post sequencing DNA analysis pipelines on public clouds in an efficient manner. Our framework implements an on-the-fly compression method of intermediate data to reduce memory requirements by up to 3x. Furthermore, it uses a superior load balancing technique to reduce accesses to the HDFS. At the same time, our framework can hide data pre-processing time by streaming and pre-processing input data while the analysis pipeline is running. As compared to the state of the art GATK best-practices pipeline, SparkGA2 is up to 22% faster on a large big data cluster of 67 nodes and up to 9% faster on a smaller cluster of 6 nodes. This indicates that SparkGA2 has better scalability characteristics compared to existing solutions, while still being able to run on a small cluster, making it suitable for practical production environments. Including the streaming solution, where data pre-processing in parallel is considered, SparkGA2 is 51% faster on a 6 node cluster. The source code of SparkGA2 is publicly available at https://github.com/HamidMushtaq/SparkGA2.
